# P31 Cogan's syndrome—a neglected autoimmune vasculitis

**DOI:** 10.1093/rap/rkab068.030

**Published:** 2021-10-19

**Authors:** Muhammad Adil Seelarbokus, Clive Kelly

**Affiliations:** South Tees Hospitals NHS Foundation Trust, Middlesbrough, United Kingdom

## Abstract

**Case report - Introduction:**

Vasculitis carries high levels of morbidity and mortality. It can affect young people where the diagnosis is often unsuspected until life-threatening complications arise. Early intervention can dramatically improve the natural history of the condition and an awareness of the clinical presentation assists clinicians in diagnosis.

Cogan’s syndrome is a rare variable-vessel vasculitis typically characterised by a non-syphilitic interstitial keratitis and vestibuloauditory symptoms, with a presumed autoimmune component. Systemic involvement may occur in up to 80% of patients. Despite over 300 cases being reported since its first description in 1934, its pathogenesis remains elusive and its diagnosis challenging.

**Case report - Case description:**

We report the case of a 27-year-old fit and well woman who was referred to ophthalmology by her GP in August 2019 due to persistent bilateral epiphora. A full ophthalmic examination revealed significant bilateral blepharitis, but was otherwise unremarkable. Notably, the cornea was clear bilaterally. She presented again to her GP in February 2020, when she developed intermittent episodes of tinnitus and vertigo which resulted in marked disability, whereby standing led to vomiting. Initial ENT review proved unavailing and no explanation was found. An MRI scan was ordered, which was normal besides a non-specific small focus of T2 hypersensitivity in the left frontal lobe.

In August 2020, she developed intermittent polyarthralgia starting with the ankles and extending proximally. Following a GP referral, she was reviewed by rheumatology in April 2021, by which point the combination of her vestibular symptoms and polyarthralgia had confined her to her house. The patient also reported daily nocturnal sweats but no weight loss. She also described a marked facial rash that extended over both cheeks down to the chin, which had resolved spontaneously and was reviewed on photographs. On examination, her lumbar spine was tender as was the ankle joint albeit no stiffness or swelling was noted. Neurological examination was normal, with no obvious hearing loss. The patient was systemically well with no evidence of cardiac involvement and no lymphadenopathy.

A full vasculitic and autoimmune screen was sent. With the exception of a mildly raised ESR and total protein, the screen came back negative.

The pattern and evolution of her symptoms led to a clinical diagnosis of Cogan’s syndrome, and treatment with prednisolone 0.5mg/kg was initiated. Her symptoms dramatically improved and dose tapering is now under way with a view of initiating mycophenolate mofetil if long-term immunosuppression proves necessary.

**Case report - Discussion:**

This case is particularly interesting as it provides valuable first-hand insight into the manifestations and diagnostic challenges that still accompany the disease, 87 years after its first appearance in the literature. It is striking to note the functional impact of the vestibular symptoms and the polyarthralgia, which confined our young, active patient to her house. This highlights the potential morbidity that can result from this disease. As remarkable as the symptoms themselves is their response to steroids, as was the case here. As such the symptoms are often manageable by the astute clinician.

Our patient’s constellation of symptoms was typical in that ocular and vestibuloauditory signs arose within the expected timeframe. However, her ocular symptoms were out of keeping with the usual features of Cogan’s syndrome, which usually comprise interstitial keratitis, conjunctivitis, uveitis, scleritis, and episcleritis. Instead, she presented with blepharitis, which had not been previously reported with Cogan’s syndrome, although there have been reports of epiphora. Moreover, it should be noted that although she had not reported hearing loss, a formal audiology assessment had not been done. Another limitation was the unavailability of inner ear auto-antibodies, which would have been helpful to consolidate the diagnostic reasoning albeit the eventual management would still be informed by clinical judgment and therefore unchanged.

Another important point that this case illustrates is the central role of GP in the diagnosis of such challenging conditions. All too often GPs are not given enough credit for the pivotal role they play in unifying the care a patient may receive from specialties that may otherwise work independently of one another. Indeed, the GP was crucial in synthesising data from different tertiary referrals and providing the key findings and the history to the rheumatologist which led to her eventual diagnosis and timely management.

**Case report - Key learning points:**

Whilst Cogan’s syndrome is now a relatively well-recognised albeit rare disease, it remains challenging to diagnose owing to its variable presentation and the lack of diagnostic tests. Consequently, its diagnosis often relies on clinical suspicion and pattern recognition. Typical Cogan’s syndrome refers to the appearance of ocular and vestibuloauditory symptoms within 2 years of each other. Systemic symptoms such as fever, weight loss, arthromyalgias, and skin rashes have all been reported. Delayed diagnosis is associated with permanent deafness and, of greater concern, a 10% risk of aortitis which may result in aortic insufficiency or dissection. This highlights the importance of prompt recognition and treatment of the disease.

A common affliction of rare diseases is the difficulty of conducting large-scale studies looking into the most effective treatment options. However, papers are frequently being published describing different approaches to the management of Cogan’s syndrome with immunosuppressants. One of the of main challenges highlighted was the management of the vestibuloauditory symptoms. Indeed, whilst ocular manifestations usually resolve with first-line steroid therapy, vestibuloauditory symptoms often require second- or even third-line treatments with immunosuppressants. Irreversible sensorineural hearing loss and persistent vestibular symptoms result in significant morbidity, and treatment aims to counter this. It has been found that initiation of high-dose steroids within 2 weeks of the onset of hearing loss was associated with the highest rates of recovery. However long-term immunosuppression is often needed to manage relapses, which occur in around 30% of patients over 10 years. This highlights the necessity for clinical trials looking into the efficacy of different immunosuppressants on patient outcomes.

As an ‘orphan disease’ with only over 300 cases reported worldwide in the last 87 years, it is a rare but reversible cause of vasculitis that all physicians should be alerted to.

